# Signals Involved in Regulation of Hepatitis C Virus RNA Genome Translation and Replication

**DOI:** 10.3389/fmicb.2018.00395

**Published:** 2018-03-12

**Authors:** Michael Niepmann, Lyudmila A. Shalamova, Gesche K. Gerresheim, Oliver Rossbach

**Affiliations:** ^1^Medical Faculty, Institute of Biochemistry, Justus Liebig University Giessen, Giessen, Germany; ^2^Faculty of Biology and Chemistry, Institute of Biochemistry, Justus Liebig University Giessen, Giessen, Germany

**Keywords:** HCV, replication, *cis*-element, untranslated region, microRNA-122

## Abstract

Hepatitis C virus (HCV) preferentially replicates in the human liver and frequently causes chronic infection, often leading to cirrhosis and liver cancer. HCV is an enveloped virus classified in the genus *Hepacivirus* in the family *Flaviviridae* and has a single-stranded RNA genome of positive orientation. The HCV RNA genome is translated and replicated in the cytoplasm. Translation is controlled by the Internal Ribosome Entry Site (IRES) in the 5′ untranslated region (5′ UTR), while also downstream elements like the *cis*-replication element (CRE) in the coding region and the 3′ UTR are involved in translation regulation. The *cis*-elements controlling replication of the viral RNA genome are located mainly in the 5′- and 3′-UTRs at the genome ends but also in the protein coding region, and in part these signals overlap with the signals controlling RNA translation. Many long-range RNA–RNA interactions (LRIs) are predicted between different regions of the HCV RNA genome, and several such LRIs are actually involved in HCV translation and replication regulation. A number of RNA *cis*-elements recruit cellular RNA-binding proteins that are involved in the regulation of HCV translation and replication. In addition, the liver-specific microRNA-122 (miR-122) binds to two target sites at the 5′ end of the viral RNA genome as well as to at least three additional target sites in the coding region and the 3′ UTR. It is involved in the regulation of HCV RNA stability, translation and replication, thereby largely contributing to the hepatotropism of HCV. However, we are still far from completely understanding all interactions that regulate HCV RNA genome translation, stability, replication and encapsidation. In particular, many conclusions on the function of *cis*-elements in HCV replication have been obtained using full-length HCV genomes or near-full-length replicon systems. These include both genome ends, making it difficult to decide if a *cis*-element in question acts on HCV replication when physically present in the plus strand genome or in the minus strand antigenome. Therefore, it may be required to use reduced systems that selectively focus on the analysis of HCV minus strand initiation and/or plus strand initiation.

## Introduction

Hepatitis C virus (HCV) preferentially infects human liver hepatocytes, likely leading to chronic liver infection and often proceeding to liver cirrhosis and hepatocellular carcinoma (HCC) (Yamane et al., 2013). Worldwide, about 70 million people are chronic carriers of HCV (WHO, 2018), and even though efficient direct-acting antivirals are now available, high treatment 

costs and emergence of resistance remain serious problems of disease control (Perales et al., 2015).

**FIGURE 1:**
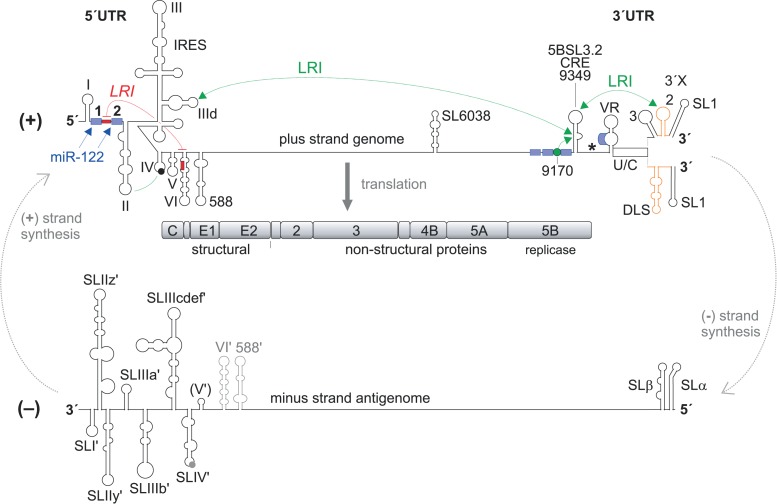
Hepatitis C virus RNA genome replication. The hepatitis C virus (HCV) (+) strand RNA genome (top) is shown with the highly structured 5′ and 3′ untranslated regions (UTRs) and additional RNA secondary structures as mentioned in the main text. The polyprotein start codon AUG is shown as filled circle in stem-loop (SL) IV of the IRES, the polyprotein stop codon as asterisk. The polyprotein is co- and post-translationally processed into the mature structural and non-structural (NS) proteins. The NS3-NS5B proteins form the replication complex with the NS5B protein as the viral RNA dependent RNA polymerase (RdRP, replicase). Stimulatory (green) and inhibitory (red) long-range RNA-RNA interactions (LRIs) and binding sites for the liver-specific microRNA-122 (miR-122) (blue boxes) are shown. The HCV genome replicates by producing a minus strand (-) antigenome (bottom), which in turn is used as the template for progeny plus strand production. RNA secondary structures are named as in the main text. CRE, *cis*-replication element, DLS, dimerization linkage sequence; IRES, internal ribosome entry site; U/C, poly(U/C) tract; VR, variable region. The drawings of the RNA secondary structures are not to scale in relation to genome length.

Hepatitis C virus comes as a lipoviral particle that contains viral proteins as well as cellular lipids and proteins, covering the viral positive-sense single-stranded RNA genome (Lindenbach, 2013; Zeisel et al., 2013). After HCV has infected the hepatocyte using a variety of entry receptors that contribute to liver specificity (Zeisel et al., 2013; Lavie and Dubuisson, 2017; Miao et al., 2017), the HCV plus strand RNA genome (**Figure 1**) is directly translated and replicated in the cytoplasm (Liu et al., 2009; Lohmann, 2013). The IRES element downstream of the 5′ end initiates polyprotein translation (Tsukiyama-Kohara et al., 1992; Niepmann, 2009b) and thereby avoids the need for a 5′ terminal cap nucleotide. This strategy allows main *cis*-elements for initiating antigenome and progeny genome RNA synthesis to reside at both very ends of the viral genome (Liu et al., 2009) (**Figure 1**). The RNA secondary structures and sequences at the genome ends are highly conserved (Fricke et al., 2015), consistent with the idea that they contain several overlapping *cis*-elements, and the roles of such *cis*-elements have been successfully analyzed using replication replicon systems that allow intracellular RNA genome amplification (Lohmann et al., 1999) as well as infectious full-length genome viral systems (Lindenbach et al., 2005; Wakita et al., 2005; Zhong et al., 2005; Pietschmann et al., 2006) (**Figure 1**).

Viral proteins which are translated from the viral genome recruit the viral RNA to the sites of replication, which are formed with membranes derived from the endoplasmic reticulum (ER) and build a so-called membranous web (Egger et al., 2002; Paul and Bartenschlager, 2013; Strating and van Kuppeveld, 2017) with vesicles that provide a protected environment for replication of the viral RNA. There, the viral RNA-dependent RNA polymerase (RdRP or “replicase”) NS5B together with other non-structural (NS) proteins of the replication complex (NS3-NS5B) uses the viral plus strand RNA genome to produce an antigenome minus strand (Lohmann, 2013) (**Figure 1**). From this minus strand, progeny plus strands are then produced in 10- to 100-fold excess over the minus strand antigenome template (Quinkert et al., 2005; Binder et al., 2007; Keum et al., 2012; Lohmann, 2013). Then, follow-up rounds of translation from the progeny plus strand genomes produce viral proteins in more than 1,000-fold excess over the viral genomes (Quinkert et al., 2005; Lohmann, 2013), and finally viral genomes are packaged in newly assembling viruses (Lindenbach, 2013; Suzuki, 2017).

Comprehensive and detailed overviews on the HCV life cycle have been previously reviewed (Liu et al., 2009; Pineiro and Martinez-Salas, 2012; Lindenbach, 2013; Lohmann, 2013; Moradpour and Penin, 2013; Niepmann, 2013; Paul and Bartenschlager, 2013; Adams et al., 2017; Gerold et al., 2017). Also the potential roles of miR-122 in the HCV life cycle have been discussed in detail (Jopling, 2008, 2012; Niepmann, 2009a; Li et al., 2013a, 2015; Wilson and Huys, 2013; Sagan et al., 2015; Sarnow and Sagan, 2016). In this review, we provide an overview on the known *cis*-elements in the viral plus strand genome and minus strand antigenome and focus on particular aspects concerning the regulation of RNA translation and RNA synthesis.

## The HCV RNA Plus Strand Genome 5′ Region

**FIGURE 2:**
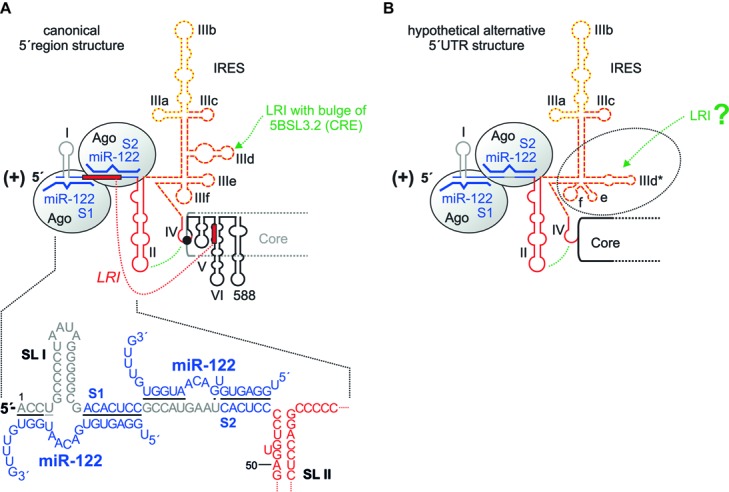
The HCV 5′ region. **(A)** The canonical HCV 5′ UTR structure with the miR-122 binding sites S1 and S2, bound by Argonaute (Ago) protein. The region of the IRES that binds the small ribosomal 40S subunit in the SL III domain with the important SL IIId is shown by a red broken line, the eIF3 binding site with the important SL IIIb with a yellow dotted line. The SL II interacts with the SL IV to place the AUG into the mRNA entry channel of the 40S ribosomal subunit. SLs V, VI and 588 are located in the core protein coding region. The seed region of miR-122 (nucleotides 2–7 or 2–8) binds to the target sequence (A)CACUCC, and the miR-122 supplementary region binds to a variable number of target nucleotides. **(B)** A hypothetical alternative structure of the IRES in which the SL IIId region is largely rearranged (Fricke et al., 2015). This predicted structure is conserved among HCV isolates and is as thermodynamically stable as the canonical structure.

The 5′ end of the plus strand (**Figure 2A**) contains highly conserved RNA secondary structures and sequences and bears *cis*-elements that control viral RNA genome translation as well as RNA replication and stability. These signals mainly reside in the 5′ UTR but also reach into the coding region of the core protein. Thereby, some of these functional signals overlap.

Translation is controlled by the IRES element (Tsukiyama-Kohara et al., 1992; Niepmann, 2009b). This comprises the stem-loop (SL) domains II, III, and IV. SL IV contains the AUG start codon for the initiation of polyprotein translation; thus, the IRES reaches into the core protein coding region. This “core” IRES is capable of driving translation in reporter RNAs that contain a reporter gene (like a luciferase gene) fused to the HCV 5′ UTR plus about 30 nucleotides of the core coding region. Several cellular proteins are recruited by the HCV 5′ UTR to facilitate HCV translation (reviewed in Niepmann, 2013); the contributions of some of these proteins to replication are discussed later in this review. In addition, some structures in the core coding region directly downstream of the IRES are involved in translation regulation, including the SLs V and VI (Vassilaki et al., 2008). Consistently, SL VI was also reported to be involved in overall HCV replication (McMullan et al., 2007), without specifying a distinct step in the viral life cycle. Thus, SL VI may be involved just in translation regulation or may even have a dual role in both translation and replication. SL 588 which is directly downstream of SL VI was also described to be involved in HCV overall replication (Pirakitikulr et al., 2016). However, we have not yet gained mechanistic insight into how these downstream elements regulate translation and/or replication and if the reverse complement of these sequences may have a function when present in the antigenome minus strand.

The central part of the IRES, the SL III, binds tightly to the small ribosomal 40S subunit (Spahn et al., 2001), with the apical loop of the small SL IIId with a GGG sequence being a main determinant of ribosome binding (Kieft et al., 2001). The translation initiation factor eIF3 mainly binds to the apical part of the SL III domain, SL IIIb (Kieft et al., 2001), and is required for subsequent steps of translation initiation (reviewed in Niepmann, 2013). The apical part of SL II interacts with the loop of SL IV and is involved in positioning the AUG start codon in the entry channel of the ribosomal 40S subunit (Filbin and Kieft, 2011; Filbin et al., 2013).

Despite this prominent role of SL II in translation, the most 5′ region of the HCV 5′ UTR including the SL I, SL II and the region between them have been reported to be involved in HCV overall RNA replication (Friebe et al., 2001; Kim et al., 2002), with a later study (Friebe and Bartenschlager, 2009) confirming these results. Notably, in all these studies the function of *cis*-elements involved in translation was uncoupled from their possible overlapping replication functions. To this end, heterologous IRES elements were employed in order to drive downstream reporter gene and/or selection marker as well as HCV non-structural protein expression in the replicon construct independent of the upstream HCV 5′ UTR sequences under investigation. Thus, the HCV sequences (SLs I and II) characterized in the above studies are concluded to be actually involved in the control of HCV RNA replication. However, strictly speaking this statement does not distinguish between elements in the annotated HCV sequence that are functional at the 5′ end of the plus strand, or as reverse complementary sequence at the 3′ end of the minus strand (**Figure 1**).

Bridging the SL I and spanning into the region between SLs I and II, two binding sites for the liver-specific miR-122 are located. The possibly multiple functions of miR-122 in HCV overall replication are discussed below. However, it should be mentioned here that a sequence at the base of SL VI in the core coding region can hybridize to a sequence overlapping with both 5′ UTR miR-122 binding sites and was reported to inhibit HCV translation (Wang et al., 2000; Kim et al., 2003) (**Figure 2A**). Single-stranded miR-122 guide strand can induce a conformational change in HCV 5′ UTR structure by displacing this inhibitory hybridization *in vitro* (Diaz-Toledano et al., 2009), whereas the displacement of this inhibitory interaction by invading miR-122 appears to be of minor importance *in vivo* (Goergen and Niepmann, 2012).

Finally, a rather hypothetical consideration applies to the structure of the HCV 5′ UTR. In a bioinformatic evaluation of conserved RNA structures in the HCV RNA genome and antigenome ends (Fricke et al., 2015), an alternative conformation of the HCV 5′ UTR was found in which the RNA secondary structure of the lower part of the SL III domain including the SL IIId is changed. This predicted structure (**Figure 2B**) is conserved among HCV isolates, and it is as thermodynamically stable as the “canonical” IRES structure (**Figure 2A**). However, all experimental data obtained so far (Niepmann, 2013; Yamamoto et al., 2014, and references therein) show the canonical IRES structure (**Figure 2A**). Thus, if the predicted alternative IRES structure forms at all in nature, it may be very short-lived and therefore yet escaped detection. Since the high degree of conservation suggests a function, we can only speculate if this alternative IRES structure may decrease the affinity of the IRES to the 40S subunit and by that may support the escape of the ribosome from the translation initiation site to proceed with translation elongation.

## The Plus Strand 3′ Region

**FIGURE 3:**
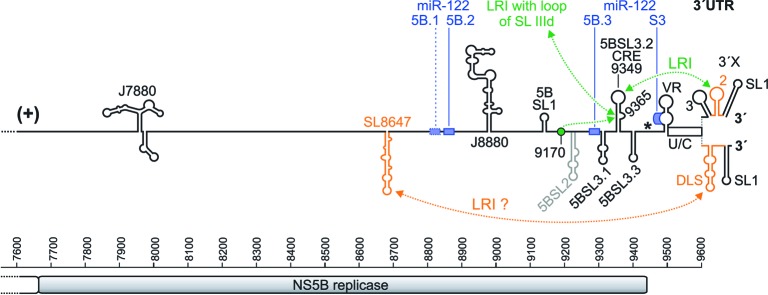
The HCV genome 3′ region. The NS5B sequence is shown with nucleotide numbers. The NS5B stop codon is shown by an asterisk, and the 3′ X region is shown in its two experimentally validated alternative structures. The miR-122 binding sites are shown as blue boxes, with the first non-conserved site 5B.1 with a dotted box and the other conserved two sites in the NS5B region (5B.2 and 5B.3) and the miR-122 target site in the 3′ UTR (S3) with solid boxes (Fricke et al., 2015). Elements suspected to be involved in RNA genome packaging are shown in orange.

The investigation of signals in the 3′ region of the plus strand RNA genome (**Figure 3**) initially focused on the 3′ UTR, although now it becomes more and more clear that several signals in the upstream coding region (**Figures 1**, **3**) are also involved in the control of HCV overall replication (Lohmann, 2013). The 3′ UTR contains a variable region (VR), a poly(U/C) tract (U/C) and a highly conserved 3′ region named the 3′ X-tail. The 3′ X and a minimal poly(U/C) tract are essential for replication, whereas the variable region is not essential but contributes to efficient replication (Tanaka et al., 1995, 1996; Kolykhalov et al., 1996, 2000; Blight and Rice, 1997; Yanagi et al., 1999; Friebe and Bartenschlager, 2002; Smith et al., 2002; Yi and Lemon, 2003a,b; Friebe et al., 2005; You and Rice, 2008). The extreme 3′-terminal nucleotides of the genome are embedded in the 3′ terminal SL 1 and contain a conserved U as terminal nucleotide (Fricke et al., 2015). This end is very sensitive to changes, and the correct end is restored in replication competent revertants after mutations had been introduced into the RNA genome (Yi and Lemon, 2003a,b). The 3′ X region can assume two major different structures. In one conformation, three stem-loops are formed, SL 1, SL 2 and SL 3 (upper structure in **Figure 3**). In an alternative conformation (lower structure in **Figure 3**) largely the SLs 2 and 3 rearrange to form one larger RNA secondary structure, while the SL 1 undergoes only a slight change at its base leading to the extrusion of two single-stranded 3′ terminal nucleotides from the double-stranded stem.

In the NS5B coding sequence, about 90 nucleotides upstream of the 3′ UTR, a sequence was characterized that forms a stem with an apical loop (position 9349) and a bulge (position 9365). This RNA element was characterized as a *cis*-replication element (CRE, also called 5BSL3.2 or SL-V) (**Figure 3**). This CRE is important for HCV replication (Lee et al., 2004; You et al., 2004), likely together with the upstream 5BSL3.1 structure (called “SL-VI” in Lee et al., 2004). However, a proposed cruciform-like structure including 5BSL3.1, 3.2 and 3.3 (You et al., 2004) is not conserved as a common structure motif among HCV isolates (Fricke et al., 2015). The bulge that is embedded in the stem of the 5BSL3.2 CRE (GCCCG) can form a long-range-interaction with an upstream sequence (CGGGC) that is located in a loosely structured region between the 5BSL1 and 5BSL2 structures (**Figure 3**) (Fricke et al., 2015). This upstream sequence is termed “9170” here (according to its approximate sequence position in the isolate JFH-1) and “9110” in Diviney et al. (2008). The interaction of the 9170 sequence with the bulge of the 5BSL3.2 CRE is essential for viral replication (Diviney et al., 2008; Tuplin et al., 2012). Mutations that modify the structure adjacent to this 9170 sequence - which may modify its exposure as a single stranded bulge – impair HCV replication (Chu et al., 2013).

In the “SL 1, 2, 3” form of the 3′ X region (upper structure in **Figure 3**), a LRI can form between the apical loop of the 3′ X SL 2 (GCUGUGA) and the apical loop of the upstream 5BSL3.2 CRE structure (UCACAGC). This “kissing-loop” interaction is required for overall HCV replication, and even covariant point mutations that retain the long-range interaction between both partners slightly reduce viral replication efficiency, pointing to the importance not only of the structure but also of the involved primary sequences (Friebe et al., 2005; You and Rice, 2008). The importance of the CRE 5BSL3.2 and the upstream 5BSL3.1 in overall replication is underlined by the fact that the NS5B replicase binds to this region as strongly as to the 3′ UTR (Lee et al., 2004).

The large RNA secondary structure formed by the combined SLs 2 and 3 (lower structure in **Figure 3**) (Fricke et al., 2015) exposes a short inverted repeat sequence (CUAG) in its apical loop. This inverted repeat loop sequence can induce dimerization of two molecules of the entire stem-loop, since two molecules of the same stem-loop can make not only intra-molecular but also inter-molecular base pairing over their stem regions. Therefore, this RNA structure has been called dimerization linkage sequence (DLS). Several studies have shown dimerization of RNAs containing the DLS sequence *in vitro* (Cristofari et al., 2004; Ivanyi-Nagy et al., 2006; Shetty et al., 2010, 2013; Cantero-Camacho and Gallego, 2015). The *in vitro* dimerization of the DLS RNA is stimulated by the HCV core protein (Cristofari et al., 2004; Ivanyi-Nagy et al., 2006; Romero-López et al., 2017), and the CRE 5BSL3.2 can interact with the 3′ X region (Shetty et al., 2013) and was shown to promote dimerization. In contrast, the IRES interferes with dimerization of the DLS (Romero-López et al., 2017). This shows that the RNA structures in the 3′ X region are highly dynamic and suggests a possible switch between a state of the HCV genome undergoing translation and/or replication and a different state of the genome that possibly dimerizes for packaging. However, evidence for an actual dimerization of complete viral genomes *in vivo* – like it has been demonstrated for human immunodeficiency virus (HIV) (Chen et al., 2009; Moore et al., 2009) – has not yet been presented for HCV.

Contradictory results have been published regarding a possible role of the CRE in the regulation of translation. One study (Tuplin et al., 2015) shows that locked nucleic acid (LNA) antisense oligonucleotides that either bind to the apical loop of the CRE or to the apical loop of the 3′ UTR SL 2 interfere with translation, suggesting that the “kissing-loop” interaction is involved in translation stimulation. This interference partially works also when the HCV IRES in the reporter constructs is replaced by the poliovirus IRES, suggesting that the stimulation of translation by the CRE-SL 2 interaction is largely independent of the nature of the 5′ UTR. Since the HCV 3′ UTR can stimulate translation in the absence of the CRE (Bradrick et al., 2006; Song et al., 2006; Bung et al., 2010), the above results suggest that the ability of the 3′ UTR to bind to the ribosomal 40S subunit (Bai et al., 2013) may be enhanced by the CRE. In contrast, another study (Romero-López and Berzal-Herranz, 2012) shows that the CRE may interfere with the stimulation of translation by the 3′ UTR. Since in the latter study a construct was used that includes NS5B sequences only starting with the 5BSL3.1, we can only speculate if upstream NS5B sequences may somehow modulate the influence of the CRE on the 3′ UTR in translation regulation.

Moreover, another important RNA secondary structure is located in the NS5B coding sequence upstream of the CRE (**Figure 3**, “SL8647”). This SL8647 element is named JFH-SL8647 in Chu et al. (2013) and J8640 in Mauger et al. (2015). It starts at position 8647 of the annotated sequence of HCV isolate JFH-1 (NCBI entry: AB047639), and its apical loop sequence (CACG) is at position 8683. This element was reported to have a role in virus production but not that much in RNA replication (Chu et al., 2013; Mauger et al., 2015), suggesting a role in late steps of the HCV life cycle like RNA packaging. Using a LRI prediction program (Fricke and Marz, 2016), we predict that a part of this SL8647 sequence (UUCACGG) interacts with part of the SL 2/DLS in the 3′ X (CUGUGAA). Since the entire 3′ X region in the 3′ UTR has been shown to contribute to RNA packaging (Shi et al., 2016), these findings suggest that the upstream NS5B sequence SL8647 may interact with the 3′ X during genome packaging.

In addition, three other distinct RNA secondary structure elements upstream of the CRE have been characterized to be involved in overall HCV RNA replication. One such element is located in the NS4B region and is termed “SL6038” (**Figure 1**); it was shown to be involved in RNA replication (Pirakitikulr et al., 2016). The second element is termed “J7880” (**Figure 3**) and also is involved in RNA replication (Mauger et al., 2015). The third element (J8880, **Figure 3**) is located even more downstream; it has an effect on early genome replication but virtually no effect on final virus particle production (Mauger et al., 2015). However, it is not yet clear if and how these sequences interact with other elements in the RNA genome and with the replication machinery.

## Long-Range Interactions Between Both Genome Ends

Interactions between both viral genome ends may serve two different purposes. On the one hand, an end-to-end interaction in *cis* may serve as an integrity control as in capped and polyadenylated mRNAs, indicating that the viral RNA is intact and thus worthy of efficient translation and replication. Accordingly, the HCV 3′ UTR stimulates translation directed by the IRES in *cis* (Ito et al., 1998; Bradrick et al., 2006; Song et al., 2006; Bung et al., 2010). All elements of the 3′ UTR contribute to this stimulation, with the SLs 2 and 3 having the mildest effect. On the other hand, also interactions that regulate a switch between translation and replication can be expected to occur between both genome ends. Mechanistically, these interactions are mediated by RNA *cis*-elements as well as by the small ribosomal 40S subunit and by *trans*-acting proteins.

There are several predicted long-range RNA–RNA *cis*-interactions between both genome ends conserved among 106 HCV isolates representative for all known genotypes (Fricke et al., 2015). These interactions could be even extended to include up to 62 nucleotides that are predicted to hybridize between the HCV 5′ UTR and 3′ UTR to form a circularized genome (Fricke et al., 2015) as reported for the Dengue Virus (Alvarez et al., 2005). However, genetic approaches to experimentally demonstrate such an interaction in a full-length genome failed (Fricke et al., 2015), most likely because any mutation in the highly conserved genome ends kills the virus and cannot be rescued by compensating mutations.

Using a newly developed “LRIscan” program, even more conserved LRIs preferentially between both HCV genome ends were predicted (Fricke and Marz, 2016). This indicates that many RNA–RNA interactions between the genome ends regulate a functional circularization of the viral RNA. In addition, RNA–RNA interactions between other genome regions may occur. While the NS5B sequences appear to be more important than other regions (Chu et al., 2013), nucleotide covariation between the 5′ UTR and the NS2/NS3 regions (Sun et al., 2011) suggests that further long-range RNA-RNA interactions may also contribute to HCV replication.

Interactions between both genome ends may also regulate a switch between translation and replication. The bulge of the CRE 5BSL3.2 in the NS5B sequence (GCCCG) can interact with the loop of the small SL IIId (UCCCU) in the large IRES SL III domain (Romero-López and Berzal-Herranz, 2009, 2012, 2017) (**Figures 1**, **3**). Since the GGG sequence in the SL IIId loop is a main determinant in the isolated IRES RNA that is required for 40S ribosome binding (Kieft et al., 2001), this suggests that the function of the SL IIId in ribosome binding and the above described interaction with the bulge of the CRE might be mutually exclusive. This CRE – SL IIId LRI is also mutually exclusive with the interaction of the sequence 9170 (9110) with the CRE bulge. Altogether, changes between these interactions are considered to regulate the switch between translation and replication (Romero-López and Berzal-Herranz, 2017), but their actual functional consequences yet need to be validated experimentally.

**FIGURE 4:**
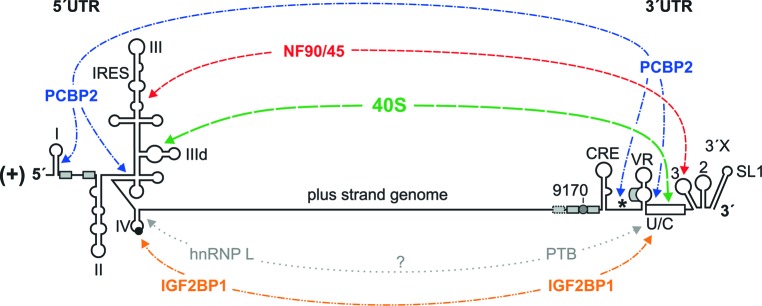
Long-range interactions of the HCV genome ends mediated by *trans*-acting factors. The small ribosomal 40S subunit interacts with the lower part of the SL III domain in the HCV 5′ UTR and with the HCV 3′ UTR, preferentially with the poly(U/C) tract (U/C). The NF90/NF45 complex interacts with the upper part of the SL III domain in the 5′ UTR and with the SL 3 in the 3′ UTR. PCBP2 interacts with each two sites in the 5′ UTR and in the 3′ UTR. IGF2BP1 (or IMP1) interacts with the 3′ region of the IRES and with the poly(U/C) tract of the 3′ UTR. A hypothetical interaction (shown by gray dotted lines) may also involve hnRNP L (which binds the 3′ region of the IRES) and PTB [which binds the poly(U/C) tract]. In addition, cooperative aggregation of miR-122/Ago complexes may also be hypothesized to contribute to the RNA end-to-end communication (not illustrated in the figure).

In addition to the *cis*-interactions described above, the 3′ UTR was found to bind to the ribosomal 40S subunit (Bai et al., 2013). Therefore, the HCV 3′ UTR also stimulates translation of RNAs that have a cap or other IRES elements at the 5′ end (Bung et al., 2010). The binding of the 3′ UTR to the 40S subunit is not seriously competed by the 5′ UTR and vice versa, indicating that 5′ UTR and 3′ UTR bind to largely different sites on the 40S subunit (Bai et al., 2013). Consistent with the finding that the poly(U/C) tract (“U/C” in **Figures 1**, **3**) is important for translation stimulation (Song et al., 2006), the poly(U/C) tract was found to be important for 40S binding (Bai et al., 2013). Supported by these interactions with the 40S subunit, HCV 5′ UTR and 3′ UTR are brought closely together, and the HCV genome ends are protected (**Figure 4**).

Moreover, also cellular RNA-binding proteins stimulate HCV genome circularization (**Figure 4**), and some have been functionally linked to HCV replication. These are insulin-like growth factor 2 mRNA-binding protein 1 (IGF2BP1 or IMP1), heterogeneous nuclear ribonucleoprotein L (hnRNP L), a complex containing the nuclear factors (NF) 90 and NF45, and poly-C binding protein 2 (PCBP2). Moreover, functional circularization of the HCV genome by binding to the ribosomal 40S subunit may also be supported by the oligomerization of Argonaute (Ago) protein which associates with miR-122 at the 5′ end and the 3′ end of the HCV RNA (see below); it can be assumed that Ago protein routinely associates with ribosomes since it was initially purified from ribosomes (Zou et al., 1998).

IGF2BP1 can bind to the HCV 5′ UTR and the 3′ UTR (**Figure 4**) and stimulates HCV translation (Weinlich et al., 2009), likely supported by the ability of the protein to dimerize (Nielsen et al., 2004). Also hnRNP L binds to the HCV IRES (**Figure 4**), and its binding has been correlated with IRES activity (Hahm et al., 1998). hnRNP L can also interact with PTB (hnRNP I) (Kim et al., 2000) which in turn binds to the poly(U/C) tract of the HCV 3′ UTR (**Figure 4**) (Ito and Lai, 1997). Thereby, also these interactions can be hypothesized to support a functional genome circularization. Moreover, the cellular RNA chaperones NF90/NF45 bind to the ends of the HCV RNA genome and facilitate genome circularization (**Figure 4**) (Isken et al., 2007). Consistent with the above considerations, both hnRNP L and NF90 have been demonstrated to stimulate HCV replication (Li et al., 2014). NF90 and NF45 stimulate the initiation of RNA synthesis of the NS5B replicase on a RNA template containing the HCV 3′ UTR including the upstream CRE region (starting from 5BSL3.1) but not on a RNA template lacking the CRE region (Schmidt et al., 2017). This confirms that the CRE region is required for efficient initiation of RNA replication at the plus strand 3′ end, and that the rearrangement of RNA–RNA interactions may be required for initiation of minus strand synthesis.

PCBP2 binds to four regions of the HCV RNA, two in the 5′ UTR and two in the 3′ region (**Figure 4**) (Flynn et al., 2015). In the 5′ UTR, the SL I – II region is the first binding region, with most prominent binding to the SL I (Fukushi et al., 2001; Wang et al., 2011), likely due to the conserved stretch of C residues in the stem of SL I (Fricke et al., 2015). The second binding site is the pseudoknot and the base of the SL III domain which also contains a stretch of four C residues in a double-stranded RNA region (Fricke et al., 2015). In the HCV 3′ region, PCBP2 binds to the very 3′ coding region of NS5B, most likely to a region directly upstream of the NS5B stop codon that is conserved to be C-rich and can form a stem (which has the NS5B stop codon in its apical loop). The second binding site in the 3′ region is in the poly(U/C) tract of the 3′ UTR (Tingting et al., 2006; Flynn et al., 2015). Since PCBP2 can multimerize (Bedard et al., 2004) and bind to single-stranded as well as to double-stranded RNA regions containing some consecutive C residues (Gamarnik and Andino, 1998; Du et al., 2007), PCBP2 dimers may support a functional circularization of the HCV genome by bringing both ends together (Wang et al., 2011).

The above described interactions of proteins with the HCV RNA genome are largely non-overlapping but appear to complement each other (**Figure 4**), indicating that they may act in concert to functionally circularize the HCV genome. These interactions suggest that there may be a regulated balance between genome translation and replication. After sufficient amounts of replication proteins have been synthesized, a switch may be required in order to reduce translation and proceed with minus strand synthesis. The above mentioned proteins may, in addition to RNA–RNA long-range interactions, be involved in the regulation of such a switch.

## Signals in the Minus Strand

The secondary RNA structures that are conserved to be formed at the 5′ end as well as at the 3′ end of the antigenome minus strand (**Figure 1**) usually do not represent exact mirror images of the structures formed by the plus strand (due to different G:U base pairs). At the 5′ end of the minus strand, the structure of the SLα is well conserved, whereas the SLβ can assume three different structures (Smith and Wu, 2004; Fricke et al., 2015). The biological relevance of these structures is not yet clear, and such possible relevance may be difficult to analyze since the counterpart of these sequences at the 3′ end of the plus strand is extremely conserved and functionally relevant.

**FIGURE 5:**
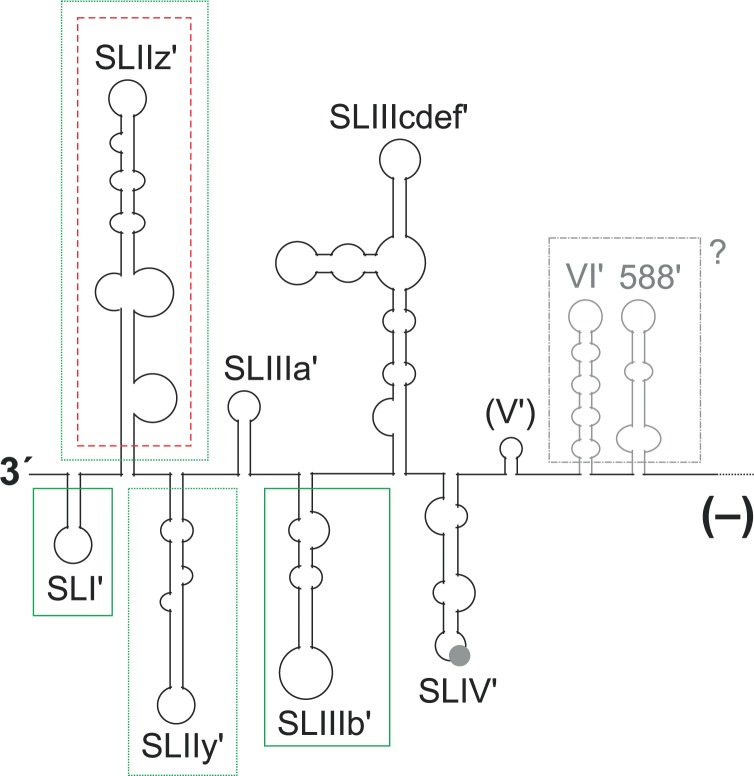
Elements in the 3′ region of the HCV minus strand antigenome involved in plus strand synthesis. The black and gray lines and structures show the 3′ region of the HCV minus strand antigenome. A contribution to plus strand initiation shown by *in vitro*-assays is indicated by boxes with solid green lines (SL I′, SL IIIb′), a negative influence indicated by these *in vitro*-assays is indicated by a box with a red broken line (SL IIz′). Sequences that were characterized by replicon studies to have a positive influence on RNA replication are boxed with dotted green lines (SL IIz′ and SL IIy′). The region which is not boxed is variable in RNA secondary structure among the predictions (see text), and no study showed a functional contribution of this region to replication. A possible relevance of the upstream structures SL VI′ and 588′ as present in the minus strand is not clear.

For the 3′ end of the minus strand (**Figure 5**), three variant structures have been proposed (Schuster et al., 2002; Smith et al., 2002; Dutkiewicz et al., 2008), of which the secondary structure of Smith et al. (2002) is the energetically most stable one and appears to be most consistent with probing data (Fricke et al., 2015). These three structures all have the most downstream five SL structures up to the SL IIIb′ as well as the SL IV′ (**Figure 5**) in common, whereas the determination of the structure of the SL III cdef′ varies among the studies. The design of local mutations for functional analyses of these sequences at the 3′ end of the minus strand was always guided by these structures. However, up to now experimental analyses were performed only in the reverse complement of the plus strand 5′ UTR but not further upstream in the minus strand. Our predictions (data not shown) of the conserved structure upstream of the SL IV′ in the minus strand using the LocARNA program (Will et al., 2012) reveal that the reverse complement sequence of the SL V in the minus strand does not form a conserved mirror of the SL V structure in the plus strand, whereas the SL VI′ and 588′ sequences form structures that roughly mirror their plus strand counterparts (gray in **Figure 5**), while having some structure changes due to different G:U base pairs.

In a genetic approach, genotype-specific chimera were generated with the HCV 5′ UTR, the 3′ UTR and the NS5B replicase either from genotype 1b (the Con1 isolate) or from genotype 2a (the JFH-1 isolate). The results showed that the genotype-specific NS5B replicase proteins appear to have preferences for their own (i.e., genotype-specific) templates at both genome ends (Binder et al., 2007). In a more detailed genetic analysis of the 3′ end of the minus strand, a replicon system was used in which the replication sequences under investigation were uncoupled at least from their translation activities by the use of a downstream IRES that drives selection marker and HCV replication protein expression (Friebe and Bartenschlager, 2009). Exchange of the G-C rich SL I′ in the minus strand by an A-U rich stem (a change that also causes a corresponding change in the SL I at the 5′ end of the opposing plus strand) abolishes HCV replication. Also modifications of the next two structures, SL IIz′ and SL IIy′, severely impaired HCV replication. In contrast, modifications of more upstream sequences had only mild effects on HCV replication in that system (Friebe and Bartenschlager, 2009). However, these studies only included 5′ UTR sequences but did not extend into the core coding region or their reverse complement like other studies did (McMullan et al., 2007; Vassilaki et al., 2008). The fact that the latter studies (McMullan et al., 2007; Vassilaki et al., 2008) utilized replicon systems, and overall genome amplification and virus production was used as a readout, leaves room for the speculation that sequences reverse complementary to the core coding region might have a role in replication even when present in the 3′ region of the minus strand (gray in **Figure 5**).

Biochemical *in vitro* approaches were used to completely uncouple the initiation of plus strand RNA synthesis at the 3′ end of the minus strand from the functions of the mirror sequences in the opposing plus strand (Astier-Gin et al., 2005; Masante et al., 2008; Mahias et al., 2010). The results of these studies are in accordance with the importance of the small 3′ terminal SL I′ for plus strand initiation. The isolated recombinant NS5B protein binds to the minus strand 3′ end RNA, and mutations in the 3′-terminal SL I′ (also called SL-A1) decrease the efficiency of plus strand synthesis. This finding is in accordance with the above findings obtained with an amplifying genome (Friebe and Bartenschlager, 2009).

In contrast, for the more upstream sequences at the 3′ end of the minus strand, these biochemical approaches returned results that are somewhat different from those obtained in the replicon studies discussed above (Friebe and Bartenschlager, 2009). In the biochemical experiments, the SL IIz′ revealed to inhibit RNA synthesis (indicated by the box with a red broken line around SL IIz′ in **Figure 5**), since it was found that its deletion resulted in enhanced RdRP activity (Astier-Gin et al., 2005). Moreover, the structure of the more upstream SL IIIb′ (SL-E1) affects the efficiency of the RdRP reaction (Astier-Gin et al., 2005; Mahias et al., 2010), while this region was reported to be of minor importance by the genetic studies (Friebe and Bartenschlager, 2009). In contrast, the even more upstream sequences of the SL VI′ and 588′ structures have not been tested *in vitro* (Astier-Gin et al., 2005; Masante et al., 2008; Mahias et al., 2010). Thus, strictly speaking we do not know yet if these sequences (McMullan et al., 2007; Vassilaki et al., 2008) are active when present in the plus strand (**Figure 1**, top) or as a reverse complement in the minus strand (**Figure 5**).

## The Liver-Specific microRNA-122

The liver-specific miR-122 (Chang et al., 2004; Jopling, 2012) initially was found to positively regulate overall HCV replication (Jopling et al., 2005). There are five to six target sites for miR-122 in the HCV RNA genome (**Figure 1**), depending on genotype (Fricke et al., 2015). Two adjacent conserved target sites (S1 and S2) are located in the 5′ UTR (Jopling et al., 2008) (**Figure 2A**). One site (5B.1) that is not conserved and two sites (5B.2 and 5B.3) that are highly conserved among HCV isolates are located in the NS5B coding region, and one conserved site (S3) is located in the otherwise variable region of the 3′ UTR (**Figure 1**). miR-122 is required for efficient overall HCV genome replication, but low-level HCV replication can occur independently of miR-122 (Thibault et al., 2013). By binding miR-122, the replicating HCV RNA sequesters miR-122 from its natural cellular mRNA targets (Luna et al., 2015), thereby possibly interfering with the fine-tuning of cellular metabolism (Krützfeldt et al., 2005; Esau et al., 2006) and the differentiation status of the hepatocytes (McGivern and Lemon, 2011).

The two binding sites in the 5′ UTR have a rather fixed distance (Fricke et al., 2015). *In vitro*, two miR-122 molecules can bind independently of each other to these sites, to the second binding site (S2) with higher affinity than to the first binding site (S1) (Mortimer and Doudna, 2013). *In vivo*, Argonaute (Ago) protein, a key component of microRNA–protein (miRISC) complexes (Schirle et al., 2014), confers binding of the two miR-122 molecules to these target sites (Roberts et al., 2011; Wilson et al., 2011; Shimakami et al., 2012a; Conrad et al., 2013). Then, binding of miR-122/Ago2 to the first binding site (S1) is stronger than to the second site S2, probably due to the fact that the target nucleotides in the HCV RNA opposite to the so-called seed sequence of the miRNA (nucleotides 2–8) are seven nucleotides in site S1 and only six nucleotides in site S2 (**Figure 2A**). In miRNA/Ago2 complexes, the seed sequence of the miRNA is primarily exposed by the Ago protein, and miRNA binding to the target RNA is initiated in this region. Therefore, this region is more important for binding target than the following 3′ nucleotides of the miRNA (Schirle et al., 2014). Continuous pairing of miR-122 to its target sites appears to be counter selected by evolution (Fricke et al., 2015), likely to avoid cleavage of fully hybridized target HCV RNA genomes by the slicer activity of the Ago2 protein (Schirle et al., 2014). The seed target nucleotides and the so-called supplementary binding region are separated by the SL I in target site 1, and by some intervening nucleotides conserved not to pair to miR-122 in target site 2. Due to the conserved proximity of both target sites in the HCV 5′ UTR, the two miR-122/Ago2 complexes bind cooperatively to these sites (Thibault et al., 2015; Nieder-Röhrmann et al., 2017). This cooperative binding may be the reason for the observation that mutations in any of these two sites generally affect the downstream effector functions of these complexes – no matter which of the different effector functions is analyzed.

The effector functions of miR-122 binding to the 5′ UTR appear to be complex and are still controversially discussed. The first studies described a strong effect of miR-122 on overall HCV replication (Jopling et al., 2005). However, at that time it was not clear which specific step in the HCV replication cycle may be affected: RNA translation, RNA synthesis, RNA genome stability and/or other steps. Later, it was shown that one such effector function of miR-122 is the stimulation of HCV translation (Henke et al., 2008), confirmed by several other studies (Bung et al., 2010; Jangra et al., 2010b; Roberts et al., 2011, 2014; Wilson et al., 2011; Fehr et al., 2012; Goergen and Niepmann, 2012; Zhang et al., 2012; Bradrick et al., 2013; Conrad et al., 2013; Huys et al., 2013; Nieder-Röhrmann et al., 2017). While the mechanisms of translation stimulation by the miR-122/Ago2 complexes binding to the 5′ UTR are not yet clear, changes in RNA template stability were shown not to be the primary cause for the effect of miR-122 on HCV translation (Henke et al., 2008; Bradrick et al., 2013; Huys et al., 2013; Roberts et al., 2014; Nieder-Röhrmann et al., 2017). A downstream function during HCV translation stimulation by miR-122 may involve the P body protein LSm1 (Roberts et al., 2014).

The second downstream effector function of the two miR-122/Ago2 complexes binding to the HCV 5′ UTR is to protect the 5′ end of the genomic RNA against degradation by 5′–3′-exonucleases (Machlin et al., 2011; Shimakami et al., 2012a,b; Li et al., 2013b; Sedano and Sarnow, 2014). The exonucleases Xrn1 (Li et al., 2013b; Moon et al., 2015; Thibault et al., 2015) as well as Xrn2 (Sedano and Sarnow, 2014) have been implicated in this process. Interestingly, early after transfection, HCV RNA is susceptible to both the 5′ exonuclease Xrn1 and to the exosome 3′ exonuclease complex. In contrast, later during HCV replication when the membranous web replication complexes have been established, HCV RNA can only be attacked by Xrn1 but not by the exosome, while miR-122 protects against this degradation (Li et al., 2013b). This points out that there may be functional differences between HCV RNA molecules contained or not contained in the protective environment of the membranous web replication complexes. In this context, it is important to note that P-body components like PatL1, LSm1, DDX3, and DDX6 are involved in HCV translation and replication (Scheller et al., 2009; Angus et al., 2010; Jangra et al., 2010a; Roberts et al., 2014). Together with other P-body components, Xrn1 is also recruited to lipid droplets upon HCV infection and co-localizes with the HCV core protein (Ariumi et al., 2011). Thus, it may be possible that Xrn1 is just accidentally co-recruited to the site of HCV replication along with other P-body components, and the protection of the HCV 5′ end by miR-122 may be a countermeasure to cope with this co-recruitment of Xrn1 to the sites of HCV replication.

The third effector function of the miR-122/Ago complexes binding to the HCV 5′ UTR is assumed in RNA replication. According to effects of miR-122 not explained by the functions actually analyzed, one study proposed a function in replication (Jangra et al., 2010b). This suspected function was shown not to occur during RNA synthesis elongation (Villanueva et al., 2010). Consistent with these ideas, Xrn1 knockdown does not rescue replication of a viral mutant defective in miR-122 binding to the 5′ UTR (Li et al., 2013b), again indicating that miR-122 may also have additional functions in the HCV life cycle. Approaching this point, a function of miR-122 in replication was proposed that could stimulate HCV RNA synthesis by altering the balance of viral RNAs engaged in replication versus translation (Masaki et al., 2015). Moreover, it has been shown that revertants in the 5′ UTR miR-122 binding sites that do not support miR-122 binding can be stable (Hopcraft et al., 2016), even suggesting a miR-122-independent role of the primary sequence of the respective HCV 5′ UTR region in the HCV life cycle. On the other hand, an decrease in cellular miR-122 concentration lead to the emergence of a mutation in the HCV sequence that slightly increased the affinity of miR-122 binding, indicating a certain need for miR-122 in efficient HCV replication (Israelow et al., 2014).

However, all above studies focused on the 5′ UTR miR-122 binding sites. In contrast, only few reports focused on the NS5B and 3′ UTR binding sites so far. Ago2 crosslink-immunoprecipitation (CLIP) experiments have shown that Ago2 binds mainly to the HCV 5′ UTR miR-122 target sites but also to the NS5B and 3′ UTR target sites (Luna et al., 2015). miR-122 (Nasheri et al., 2011) as well as miR-122/Ago2 complexes (Gerresheim et al., 2017) bind to these sites, while binding strength depends on local RNA structure, which may change depending on local RNA function, e.g., during translation (Gerresheim et al., 2017). Contradictory results have been published about possible roles of the NS5B and 3′ UTR miR-122 binding sites in regulation of translation and replication (Henke et al., 2008; Nasheri et al., 2011; Gerresheim et al., 2017). Thus, possible functions of the downstream miR-122 binding sites need further evaluation, likely requiring specialized analysis systems that each focus on one particular effector function.

Other putative functions of miR-122 in the HCV replication cycle have not yet been resolved. It has been speculated that miR-122 affects the switch from translation to replication, the displacement of the plus strand RNA from its minus strand template following replication, the modulation of binding of RNA binding proteins and the recruitment of the HCV RNA to replication complexes (Sarnow and Sagan, 2016).

## Concluding Remarks

Even though much progress has been achieved in the analysis of the molecular biology of the HCV life cycle, there are still several open questions: (1) Which signals and *trans*-acting factors are involved in the initiation of minus strand synthesis? (2) Do plus strand 5′ region sequences regulate minus strand initiation at the genome’s 3′ end? (3) Does translation at the genome 5′ end interfere with minus strand initiation or vice versa? (4) What are the molecular details of the initiation of plus strand synthesis? (5) Do the complete HCV genomes actually dimerize *in vivo*? (6) Which determinants of the HCV RNA genome are involved in packaging of progeny genomes? (7) How is the stimulation of translation by miR-122 conferred? (8) Are there additional roles of the 5′ UTR miR-122 binding sites in replication? (9) What are the roles of the NS5B and 3′ UTR miR-122 binding sites? These and other questions need to be answered in future studies.

## Author Contributions

MN: writing the original draft. MN, LS, GG, and OR: reviewing and editing the manuscript. MN and OR: funding acquisition.

## Conflict of Interest Statement

The authors declare that the research was conducted in the absence of any commercial or financial relationships that could be construed as a potential conflict of interest.
